# Sex Differences in Adult Incarceration After Pediatric Traumatic Brain Injury

**DOI:** 10.1089/neur.2023.0066

**Published:** 2024-04-11

**Authors:** Anurag Modak, Kyle E. Zappi, Alexander J. Catoya, Mehdi S. Lemdani, Gretchen M. Koller, Laurel Seltzer, Ryan E. Radwanski, Susan C. Pannullo

**Affiliations:** ^1^ Department of Neurosurgery, New Jersey Medical School, Robert Wood Johnson Medical School, Rutgers University, Newark, New Jersey, USA.; ^2^ Center for Advanced Biotechnology and Medicine, Robert Wood Johnson Medical School, Rutgers University, Newark, New Jersey, USA.; ^3^ Brain and Spine Group, Bridgewater, New Jersey, USA.; ^4^ Department of Neurological Surgery, Joan & Sanford I. Weill Medical College, College of Engineering, Cornell University, New York, New York, USA.; ^5^ College of Medicine, Kansas City University, Kansas City, Missouri, USA.; ^6^ Tulane University School of Medicine, New Orleans, Louisiana, USA.; ^7^ Department of Biomedical Engineering, College of Engineering, Cornell University, New York, New York, USA.

**Keywords:** brain injury, incarceration, pediatric traumatic brain injury, sex differences

## Abstract

Pediatric traumatic brain injury (pTBI) is a major risk factor associated with adulthood incarceration. Most research into the link between pTBI and adulthood incarceration has focused on incarcerated males, who comprise the vast majority of incarcerated adults, particularly in industrialized nations. In this review, we sought to identify sex-related differences in the incidence and pathophysiology of pTBI and subsequent risk of adulthood incarceration. A scoping review was undertaken using PubMed, Scopus, Ovid, and the Cochrane Library. Articles analyzing sex-related differences in pTBI and adult incarceration rates, studies conducted on an incarcerated population, and cohort studies, cross-sectional studies, clinical trials, systematic reviews, or meta-analyses were included in this review. Of the 85 unique results, 25 articles met our inclusion criteria. Male children are 1.5 times more likely to suffer a TBI than females; however, the prevalence of incarcerated adults with a history of pTBI is ∼35–45% for both sexes. Neurophysiologically, female sex hormones are implicated in neuroprotective roles, mitigating central nervous system (CNS) damage post-TBI, although this role may be more complex, given that injury severity and sequelae have been correlated with male sex whereas increased mortality has been correlated with female sex. Further investigation into the relationship between estrogen and subsequent clinical measurements of CNS function is needed to develop interventions that may alleviate the pathophysiological consequences of pTBI.

## Introduction

The incidence of pediatric traumatic brain injuries (pTBIs) is linked to adult incarceration. Children suffering from traumatic brain injury (TBI) possess a significantly increased relative risk of incarceration by adulthood.^1–4^ The pathway from pTBI to adult incarceration is complex and mediated by numerous biological, psychological, and social variables.^5^ Briefly, post-concussive attentional and executive function deficits and decreased neural efficiency contribute to increased fatigue and sleep disturbances and impaired information recognition, encoding, and retrieval. These changes yield impairments in academic skills and social and emotional intelligence, increased impulsivity and decreased self-regulation, problems with attention, poor decision making, increased sensation seeking, and increased risk-taking behavior such as antisocial behavior and criminality.^4,6^ Other neuropsychiatric consequences include oss of pragmatic communication ability in the written and spoken domains, which diminishes the capacity of juveniles to effectively express themselves to officers of the criminal justice system.^7^ These juveniles thus encounter challenges when interacting with law enforcement, serving to increase their risk of incarceration.^7^

However, much of the research in this area has focused on incarcerated males, who comprise the vast majority of incarcerated adults, particularly in industrialized nations.^1–4,8^ A gap exists in our understanding of the pathway from pTBI to adult incarceration among the female population because of limited available literature and the poor generalizability of data collected on incarcerated males.^9–13^ Several biological, psychological, and social determinants may account for the observed differences; data on these issues are emerging.^11^

One of the more salient questions in this area, from a neurotrauma and neurocritical care perspective, is why are young females who experience TBI less likely to become incarcerated as adults when compared to their male counterparts? Current data on the incidence of TBI among incarcerated adults already demonstrates a lower incidence of TBI among incarcerated females, and this discrepancy is further compounded by the fact that females are less likely to become incarcerated overall.^8–13^ Herein lies the gap in our current understanding as well as the opportunity to generate tangible, beneficial changes in the clinical approach to pTBI.

The known data are suggestive of some neurophysiological differences in the response to pTBI between males and females; however, the precise differences are poorly elucidated. Understanding these differences will enable clinicians to develop better clinical measurements of central nervous system (CNS) function that stratify participants by age and sex and develop interventions that may alleviate the pathophysiological consequences of pTBI. Therefore, it is essential to understand the reason for how and why the pathway from pTBI to adult incarceration progresses differently in females and highlight the sex-based differences. In this review, we sought to identify sex-related differences in the incidence and pathophysiology of pTBI and subsequent risk of adulthood incarceration.

## Methods

A scoping review was performed according to the Preferred Reporting Items for Systematic Reviews and Meta-Analyses Extension for Scoping Reviews (PRISMA-SR) guidelines to investigate sex-related differences in pTBI and adult incarceration rates.^14^ We determined that performing a scoping review was most appropriate given the broad nature of our research and our aim of summarizing and synthesizing the existing literature on the sex-based differences in neurological outcomes after pTBI, incidence of pTBI, and adulthood criminality and incarceration rates among persons with a history of pTBI.

A keyword search was conducted on PubMed, Scopus, Ovid, and the Cochrane Library. Articles were selected for inclusion if the titles and/or abstracts mentioned sex-related differences in pTBI and adult incarceration rates; if the studies were conducted on an incarcerated population; and if they were cohort studies, cross-sectional studies, clinical trials, systematic reviews, or meta-analyses. Articles were excluded if they were published before the year 2000.

After reviewing the literature, the sex-based neurobiological mechanisms, pTBI incidence differences, and predisposing factors that mediate sex-related differences in pTBI and adult incarceration rates were identified. For each mechanism or associated change, the quality of the evidence was assigned a classification based on the five-level system reported by Rothoerl and colleagues and Yarascavitch and colleagues in their systematic reviews of the quality of evidence in neurosurgical publications.^15,16^ This classification system is ultimately derived from the guidelines published by the Oxford Center for Evidence-Based Medicine.^17^

## Results

**FIG. 1.**
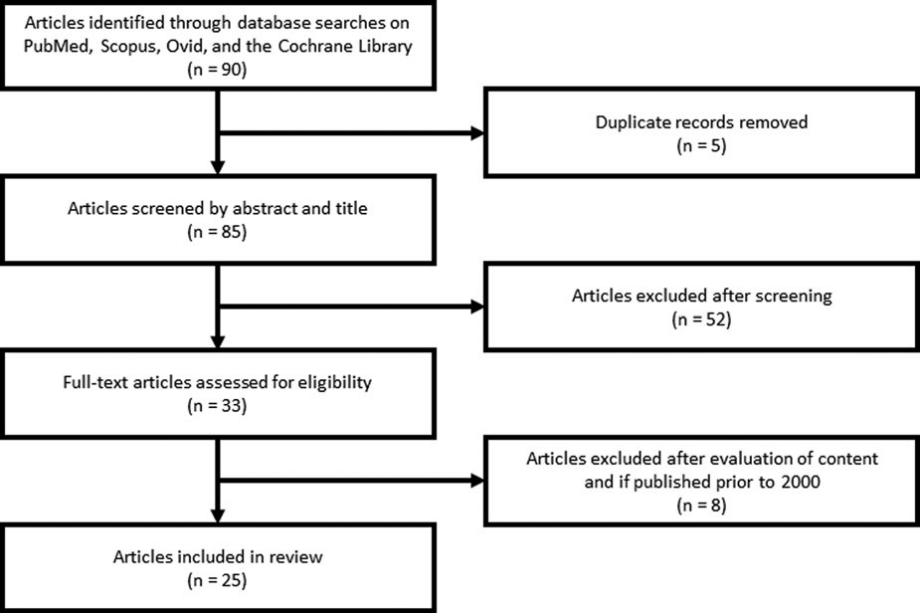
Schematic diagram showing the PRISMA-SR process for screening search results. Narrowed results included 25 articles in the final review.

**FIG. 2.**
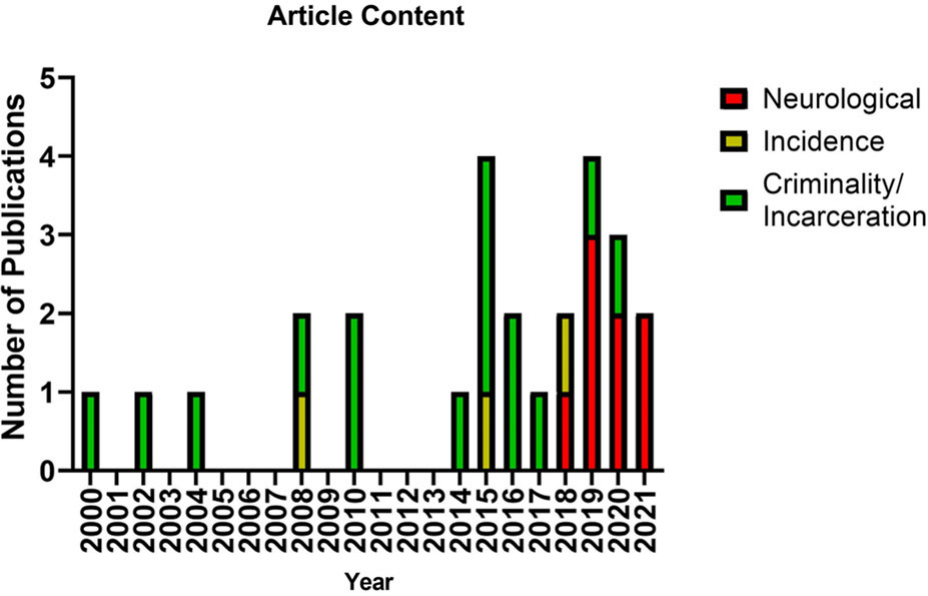
Graph depicting the distribution of included articles based on category of content matter with respect to time.

A total of 90 articles were retrieved, of which five were duplicate results. All of the 85 unique results were subjected to title/abstract screening and 25 articles met our inclusion criteria after a full-text review (Fig. 1). An analysis of the content of each article revealed that eight articles focused on sex-based differences in post-TBI neurological mechanisms, three on sex-based differences in the incidence of pTBI, and 15 on the factors associated with sex-based differences in adulthood criminality and incarceration rates among persons with a history of pTBI (Fig. 2). Several articles dealt with more than one class of variable of interest. Articles were published between January 1, 2000 and December 31, 2021. The majority of articles were published after 2014.

We identified several variables associated with sex-based differences in adulthood criminality and incarceration rates among persons with a history of pTBI. Articles included in our review were predominantly systematic reviews and cohort studies, so the highest level of evidence rating awarded to any specific factor was IIA.^15,16^ Levels of evidence classifications associated with each identified factor are listed in Table 1.

**Table 1.** Sex-Related Factors Associated With the Pre-pTBI Setting and/or Post-pTBI Course, General Effect of Each Associated Factor, and Level of Evidence in Support of the Association

Factor	Effect	Level of evidence
Male sex	Associated with increased risk of suffering TBIs	IIA
	Associated with worsening of primary ADHD post-TBI	IIIA
Estrogen	Associated with increased injury severity	IIIB
	Associated with improved healing post-TBI	IIIB

pTBI, pediatric TBI; TBI, traumatic brain injury; ADHD, attention-deficit hyperactivity disorder.

## Discussion

### Neurological effects by sex

TBI in the pediatric brain exhibits certain sex-related differences, although the mechanisms have yet to be fully elucidated.^18–22^ In both males and females, severe pTBI is associated with a significantly greater lifetime risk of impaired neuropsychological functioning and sociobehavioral problems than is mild pTBI.^1,4,6,7,23^ Still, even untreated mild pTBI can lead to attentional deficits and impaired inhibitory control, among other neurodevelopmental issues.^1,4,6,7^ Moreover, the existing literature suggests that a complex set of interactions between steroid sex hormones, neurodevelopmental stage, and psychosocial factors may underlie some observed differences.^21^ However, there appears to be little consensus regarding the specifics of these sex-related differences beyond the following generalizations: 1) male sex is associated with worse neuropathological progression and 2) female sex, perhaps through estrogen, is associated with neuroprotective responses.^18,21^ It is worth mentioning that the literature identifies several nuances, but the evidence is still limited.^18–21^

One particularly interesting avenue of research involves the role of estrogen in the post-pTBI setting. This regulatory steroid hormone reduces cerebrovascular reactivity, leading to decreased cerebral perfusion pressure and improved healing post-TBI.^21^ It also affects anatomical changes, predisposing persons to worsened impact severity and cerebral injury, particularly through decreased neurocranium thickness in females.^21,24^ Estrogen also plays a role in tissue remodeling of white matter, specifically through changes in axonal ultrastructure that increase vulnerability to shear deformation in acceleration brain injuries.^21^ However, differences in levels of sex steroid hormone levels in pediatric patients become significant only during adolescence, although these hormone play important neurodevelopmental roles *in utero*.^21^ Accordingly, it follows that the effects of estrogen possess a temporal component for which current studies do not adequately account. In short, the role of estrogen is complex and nuanced, and gaps in our current understanding may be attributable to the paucity of sufficiently powerful studies on this topic.^21,24^

Another notable area of research is the study of attention issues in the post-pTBI setting. A well-known complication of pTBI is secondary, or post-injury, attention-deficit hyperactivity disorder (SADHD), which represents significant impairment that can be suffered for several years after the inciting injury, resulting in long-term functional impairment.^1,22,25,26^ Studies on SADHD have variously found that male sex is associated with an increased likelihood of developing attentional impairments and that there are no sex-related differences in the risk of developing such neurocognitive issues.^25^ In contrast, studies on pediatric populations with primary, or pre-injury, attention-deficit hyperactivity disorder (ADHD) demonstrated that male sex is a major risk factor for worsening of primary ADHD severity in the post-pTBI setting.^22,25^ In essence, the paucity of concrete trends regarding the sex-related differences in post-pTBI events may be attributable to limitations related to sex bias with a male predominance and insufficient protocols in studies to draw powerful conclusions.^21,25,27^

### Incidence of pediatric traumatic brain injury by sex

Examination of the role of pTBI in incarcerated persons has exposed differences in the incidence of TBI in male and female youth. The annual incidence of TBI was reported as 195 per 100,000 person-years among females and 388 per 100,000 person-years among males.^10^ Male children are 1.5 times more likely to suffer a TBI than female children.^13^ Male sex is characterized as an identifiable risk factor for TBI.^13^

This correlation between incidence of TBI and sex has been underscored in various settings, including population-based studies of affected youth, as well as studies specific to incarcerated persons.^1,3,13^ One study examining TBI in a population of incarcerated adult females reported 94.7% of the cohort as having a history of TBI, with the additional increase in mental health impacts correlated with a younger age of initial TBI.^9^ Such findings demonstrate the burden of past TBI in female youth in the context of incarcerated females. These data strengthen the reciprocity between history of TBI in both male and female children and incarceration rates, underlining the significant effect of brain trauma on emotional development and brain malleability in the pediatric population as a whole.

### Incarceration and criminality by sex

Risk-taking behavior predisposes persons to injury, including TBI, while also correlating with criminal behavior.^28^ However, the increase in aggression from pre-TBI to post-TBI also suggests a potential causative factor, suggesting TBI as a potential cause for the differences in aggression between injured and uninjured populations.^28^ Data on juvenile offenders have shown that those who sustained a TBI during childhood or adolescence were more likely to partake in delinquent behavior and physical altercations, bully other children, and show increased levels of aggression compared with their uninjured counterparts.^29,30^ This relationship has proven difficult to elucidate because of multiple potential confounding variables, including socioeconomic status and past related physical abuse, complicating analysis. Morgan and colleagues note that damages to executive function may correlate with elements of psychopathy, though not psychopathy itself, particularly by damaging the cognitive ability to empathize and encouraging impulsive behavior.^23^ This relationship suggests a potential explanation for the relationship between pTBI and antisocial behavior leading to incarceration, specifically through impaired executive functioning resulting from frontal lobe damage.

These same cohorts were also more likely to report any substance use, meet criteria for alcohol or drug abuse, meet criteria for alcohol or drug dependence, and receive higher scores when measuring psychopathy, moral disengagement, and impulsivity.^29,30^ In determining whether delinquent behavior and high levels of aggression exhibited by this population were attributable to or the cause for sustaining a pTBI, Cole and colleagues found that delinquent children tend to acquire TBI from sports, falls, motor-vehicle accidents, and fights whereas non-delinquent children tend to acquire TBI from just sports, suggesting that TBI may be the result of and not a cause of delinquent behavior. Additionally, they showed that aggressive behavior increased in children from pre- to post-injury.^28^ This again suggests TBI as a potential cause for the differences in aggression noticed between injured and non-injured cohorts.^28^

Studies note a correlation between the incarcerated population and increased TBI prevalence. The prevalence of incarcerated adults with a history of pTBI is ∼35–45% for both sexes.^31^ Shiroma and colleagues note that juvenile offenders have TBI prevalence ranging from 4% to 74%, with the majority of studies showing a disproportionately higher percentage than the general population prevalence of 8.5%.^31^ Similar studies on prevalence, though varying widely because of small sample sizes at individual sites, also note that TBI prevalence is disproportionately higher among juvenile offenders than the general juvenile population.^1,32^ The incarcerated population in general also shows increased TBI prevalence, though not all of these TBIs were incurred during childhood.^33^ However, TBI offenders typically enter the prison system earlier than non-TBI offenders, at ∼16.4 years old compared to 20.1 years.^33^ This result suggests that pTBI in particular predisposes offenders to earlier criminal behavior. Sex differences in the incarcerated population suggest that males typically have a higher prevalence of TBI to females, though this difference may not be statistically significant.^31^ However, little to no literature appears to examine sex differences in criminal behavior after pTBI.

## Conclusion

Ultimately, we find that there is no significant difference in the prevalence of incarcerated adults with a history of pTBI between the two sexes despite level IIA evidence supporting the fact that male children are 1.5 times more likely to suffer a TBI than females. Our previously described biopsychosocial model predicts that a greater proportion of males than females with a history of pTBI should be incarcerated, so this apparent contradiction suggests a deficiency of data in this field regarding the differential impact of female sex on the progression from pTBI to adulthood incarceration. Neurophysiologically, female sex hormones are implicated in neuroprotective roles, mitigating CNS damage post-TBI, although this role may be more nuanced, given that injury severity and sequelae have been correlated with male sex whereas increased mortality has been correlated with female sex. However, this role is complicated by the fact that differences in pediatric steroid sex hormone levels become physiologically significant only during adolescence, so the age at which a child suffers a TBI must also be taken into account. Further investigation into the relationship between estrogen and subsequent clinical measurements of CNS function that stratify participants by age and sex are needed so that interventions that may alleviate the pathophysiological consequences of pTBI can be developed in the future.

## Transparency, Rigor, and Reproducibility Summary

The authors acknowledge the importance of transparency, rigor, and reproducibility in scientific research, and this statement outlines the measures taken to adhere to the highest standards in the present study. The authors provided a detailed description of the study design, including the research question, objectives, and hypotheses, in the article with clear delineation of specific methodology to enable other researchers to replicate our work. The data and materials used in the study are listed by the authors and are openly available. The authors adhered to all relevant guidelines and regulations pertaining to database searches, literature reviews, and ethical considerations and disclosed any potential conflicts of interest. The authors welcome collaboration, scrutiny, and replication attempts from the scientific community to further validate and build upon these findings.

## Abbreviations Used

ADHD: attention-deficit hyperactivity disorder
CNS: central nervous system
pTBI: pediatric traumatic brain injury
SADHD: secondary attention-deficit hyperactivity disorder
TBI: traumatic brain injury
